# Inclusion of Race and Ethnicity With Neighborhood Socioeconomic Deprivation When Assessing COVID-19 Hospitalization Risk Among California Veterans Health Administration Users

**DOI:** 10.1001/jamanetworkopen.2023.1471

**Published:** 2023-03-03

**Authors:** Michelle S. Wong, Arleen F. Brown, Donna L. Washington

**Affiliations:** 1 Veterans Affairs (VA) Health Services Research and Development Center for the Study of Healthcare Innovation, Implementation and Policy, VA Greater Los Angeles Healthcare System, Los Angeles, California; 2 Division of General Internal Medicine and Health Services Research, Department of Medicine, David Geffen School of Medicine at University of California, Los Angeles (UCLA); 3 Olive View–UCLA Medical Center, Sylmar, California

## Abstract

**Importance:**

Despite complexities of racial and ethnic residential segregation (hereinafter referred to as *segregation*) and neighborhood socioeconomic deprivation, public health studies, including those on COVID-19 racial and ethnic disparities, often rely on composite neighborhood indices that do not account for residential segregation.

**Objective:**

To examine the associations by race and ethnicity among California’s Healthy Places Index (HPI), Black and Hispanic segregation, Social Vulnerability Index (SVI), and COVID-19–related hospitalization.

**Design, Setting, and Participants:**

This cohort study included veterans with positive test results for COVID-19 living in California who used Veterans Health Administration services between March 1, 2020, and October 31, 2021.

**Main Outcomes and Measures:**

Rates of COVID-19–related hospitalization among veterans with COVID-19.

**Results:**

The sample available for analysis included 19 495 veterans with COVID-19 (mean [SD] age, 57.21 [17.68] years), of whom 91.0% were men, 27.7% were Hispanic, 16.1% were non-Hispanic Black, and 45.0% were non-Hispanic White. For Black veterans, living in lower-HPI (ie, less healthy) neighborhoods was associated with higher rates of hospitalization (odds ratio [OR], 1.07 [95% CI, 1.03-1.12]), even after accounting for Black segregation (OR, 1.06 [95% CI, 1.02-1.11]). Among Hispanic veterans, living in lower-HPI neighborhoods was not associated with hospitalization with (OR, 1.04 [95% CI, 0.99-1.09]) and without (OR, 1.03 [95% CI, 1.00-1.08]) Hispanic segregation adjustment. For non-Hispanic White veterans, lower HPI was associated with more frequent hospitalization (OR, 1.03 [95% CI, 1.00-1.06]). The HPI was no longer associated with hospitalization after accounting for Black (OR, 1.02 [95% CI, 0.99-1.05]) or Hispanic (OR, 0.98 [95% CI, 0.95-1.02]) segregation. Hospitalization was higher for White (OR, 4.42 [95% CI, 1.62-12.08]) and Hispanic (OR, 2.90 [95% CI, 1.02-8.23]) veterans living in neighborhoods with greater Black segregation and for White veterans in more Hispanic-segregated neighborhoods (OR, 2.81 [95% CI, 1.96-4.03]), adjusting for HPI. Living in higher SVI (ie, more vulnerable) neighborhoods was associated with greater hospitalization for Black (OR, 1.06 [95% CI, 1.02-1.10]) and non-Hispanic White (OR, 1.04 [95% CI, 1.01-1.06]) veterans.

**Conclusions and Relevance:**

In this cohort study of US veterans with COVID-19, HPI captured neighborhood-level risk for COVID-19–related hospitalization for Black, Hispanic, and White veterans comparably with SVI. These findings have implications for the use of HPI and other composite neighborhood deprivation indices that do not explicitly account for segregation. Understanding associations between place and health requires ensuring composite measures accurately account for multiple aspects of neighborhood deprivation and, importantly, variation by race and ethnicity.

## Key Points

**Question:**

How is exclusion of racial and ethnic segregation from California’s Healthy Places Index (HPI), a composite measure of the neighborhood environment, associated with race and ethnicity–stratified COVID-19–related hospitalization?

**Findings:**

In this cohort study of 19 495 veterans with COVID-19 residing in California, COVID-19–related hospitalization was higher among Black and White veterans living in neighborhoods with lower HPI scores. However, accounting for Black segregation eliminated HPI’s hospitalization association for White veterans, but not for Black veterans.

**Meaning:**

These findings suggest that care should be taken when using composite neighborhood deprivation indices that do not account for racial and ethnic segregation, as these associations may differ by race and ethnicity.

## Teaser

This cohort study examines the associations among the Healthy Places Index, Black and Hispanic segregation, and the Social Vulnerability Index with COVID-19–related hospitalization by race and ethnicity group among veterans with COVID-19 in California.

## Introduction

The neighborhood context has contributed to racial and ethnic health disparities for numerous health conditions, including COVID-19.^1,2,3,4^ Racial and ethnic residential segregation (hereinafter referred to as *segregation*) is a well-characterized determinant of racial and ethnic health disparities.^5^ While racially and ethnically segregated neighborhoods are often socioeconomically deprived, segregation also negatively affects health through other pathways.^6^ Measuring neighborhood context, particularly in single composite indices, is complicated and may not adequately account for segregation’s unique contribution to health.

Segregated neighborhoods result from entrenched structural racism within US political and social systems. Underinvestment in these communities, including policies designed to divert needed resources from these neighborhoods, leaves segregated neighborhoods with fewer social and economic resources than predominately White neighborhoods.^6,7^ Black segregated and predominately White neighborhoods of the same socioeconomic status (SES) can differ. For example, compared with predominately White neighborhoods of similar SES, Black segregated neighborhoods are overpoliced and oversurveilled, yet have fewer emergency services.^8^

Living in segregated neighborhoods may also confer social benefits for some racial and ethnic groups. For example, recent Hispanic and Asian immigrants may choose to live in “ethnic enclaves,” or neighborhoods with a high concentration of individuals of the same race or ethnicity. Some studies suggest protective effects of living in ethnic enclaves by providing residents with access to culturally familiar foods and social ties.^9^

Despite the complexities of segregation, public health studies, including those on racial and ethnic disparities in COVID-19 often rely on composite neighborhood indices that do not account for segregation.^10,11,12^ The California Healthy Places Index (HPI), for example, captures multiple neighborhood characteristics into a single score estimating health and life expectancy.^13,14^ The California HPI is a publicly available tool widely used to prioritize resource allocation and programs. Excluding race and ethnicity from HPI complies with California’s 1996 ballot proposition 209 prohibiting policy makers from using race or ethnicity to make state program funding decisions, so policy makers can use HPI to guide policy and funding allocation decisions. However, it is unclear whether excluding neighborhood racial and ethnic composition accurately captures health risk, particularly for members of racial and ethnic minority groups. In contrast, other composite indices, such as the Social Vulnerability Index (SVI), a multidimensional neighborhood socioeconomic deprivation index, account for neighborhood racial and ethnic composition.^15^ Comparing associations between COVID-19–related adverse outcomes and HPI, segregation, and indices like the SVI that account for racial and ethnic neighborhood composition can provide insight into how well these measures account for both neighborhood socioeconomic deprivation and effects of structural racism arising through segregation.

The California Department of Public Health (CDPH) adapted HPI to facilitate equity in COVID-19 vaccine distribution, pandemic-related capacity investment, and economic reopening.^16,17^ Recognizing racism’s detrimental effect on health, the CDPH analyzed the association of HPI and race and ethnicity with COVID-19 mortality. While HPI accounted for significant COVID-19 mortality, race and ethnicity independently contributed to mortality, particularly among Native Hawaiian or other Pacific Islander individuals.^18,19^ We build on this finding to understand how excluding segregation affects HPI’s association with COVID-19 severity—specifically hospitalization—by race and ethnicity.

We examined the associations of HPI, Black and Hispanic segregation, and SVI with COVID-19–related hospitalization by racial and ethnic group among veterans with positive test results for COVID-19. This allowed us to examine neighborhood associations with clinical severity of COVID-19. Our sample of Veterans Health Administration (VHA) users living in California takes advantage of data from a highly racially and ethnically diverse segment of the population using the US’s largest integrated health care system. We hypothesized that while HPI would be associated with COVID-19–related hospitalization for veterans with COVID-19, using SVI or accounting for both HPI and residential racial and ethnic segregation simultaneously would have a stronger association with hospitalization than would HPI alone.

## Methods

The Institutional Review Board of the Veterans Affairs (VA) Greater Los Angeles Healthcare System approved this cohort study and granted a waiver of informed consent of study participants for the use of deidentified data. This study followed the Strengthening the Reporting of Observational Studies in Epidemiology (STROBE) reporting guideline.

### Data and Sample

Veterans’ data came from the VHA COVID-19 Shared Data Resource, a national data set created by the VHA in response to the pandemic containing extensive demographic, clinical, laboratory, and clinical outcome data from multiple validated sources on VHA users with confirmed COVID-19 infections.^20,21^ Our sample includes veteran VHA users living in California with a positive polymerase chain reaction COVID-19 test result within the VHA or a VHA clinical note confirming diagnosis from testing outside the VHA (N = 19 495), excluding veteran VHA employees. If veterans had multiple positive COVID-19 test results, we used the date of the first test. We excluded veterans with unknown race or ethnicity (3.6% of sample). Neighborhood socioeconomic status data came from the HPI, version 3.0,^22^ and the 2018 SVI^15^ data sets, and segregation data were from the 2015 to 2019 American Community Survey 5-year estimates.^23^ Veterans’ residential addresses were geocoded to latitude and longitude coordinates, then assigned to census tracts, which we linked to HPI, SVI, and US Census data. All missing geocoding was due to missing addresses (6.1% not hospitalized; 3.7% hospitalized; 5.7% total).

### Variables

Our key dependent variable was a dichotomous indicator of whether or not veterans with COVID-19 had a COVID-19–related hospitalization (tested or treated at VHA facilities for known or probable COVID-19). We had 4 independent variables describing census tract–level neighborhood characteristics: HPI percentile,^24^ Black segregation, Hispanic segregation, and SVI percentile. The HPI, developed based on a social determinants of health framework, is a composite score created from US Census measures related to the economy, education, health care access, housing, neighborhoods, clean environment, transportation, and social environment. The SVI was created from 15 US Census variables measuring SES, household composition, race and ethnicity, language composition, housing, and transportation availability. Composite HPI and SVI scores were converted to percentiles: higher HPI percentiles indicate healthier census tracts, while higher SVI percentiles indicate greater social vulnerability. To aid in interpretating regression results, we scaled HPI and SVI percentiles to increments of 10–percentile point increases and reverse coded HPI such that odds ratios (ORs) greater than 1.00 indicate increased risk.

While multiple segregation measures exist, we used the isolation index, which captures the degree to which a member of one racial or ethnic group is exposed only to individuals of that same group.^25^ This measure has several advantages: it better captures underlying processes through which segregation can negatively affect health (eg, concentrated disadvantage) and is one of the most commonly used segregation indices.^26,27^ The isolation index is often calculated at the county level, but since some of California’s large and highly diverse counties include both segregated and integrated areas, we created separate Black and Hispanic isolation indices at the census tract level based on the 1988 formula from Massey and Denton^28^ (eMethods in Supplement 1). Isolation indices ranged from 0 to 1; larger values indicated greater segregation.

We stratified our analysis based on veteran race and ethnicity so that we could examine HPI and segregation associations by race and ethnicity. Veterans who self-identified as Hispanic or Latino were categorized as Hispanic, and all other veterans were categorized by self-identified race. We included the following race groups: American Indian or Alaska Native, Asian, Hispanic, Native Hawaiian or other Pacific Islander, non-Hispanic Black (hereinafter referred to as *Black*), and non-Hispanic White (hereinafter referred to as *White*). Control variables included age (<60, 60-64, 65-69, 70-74, 75-79, and ≥80 years), sex, individual SES (based on VA priority group^29^: high income, low income, and indeterminate income or service connected), a prior diagnosis of a comorbidity or condition identified by the Centers for Disease Control and Prevention as increasing risk for serious COVID-19 (cancer, chronic lung disease, dementia, types 1 and 2 diabetes, heart disease, HIV, liver disease, overweight or obesity, pregnancy, sickle cell anemia, ischemic stroke, cerebrovascular disease, and alcohol and drug use disorders), period of COVID-19 diagnosis (early pandemic [March 1 to May 31, 2020], summer surge [June 1 to July 31, 2020], fall drop [August 1 to October 31, 2020], winter surge [November 1, 2020, to February 28, 2021], vaccine available [March 1 to June 30, 2021], and Delta surge [July 1 to October 31, 2021]), and vaccination status (unvaccinated, fully vaccinated [2 messenger RNA or 1 adenovirus vaccine] before COVID-19 diagnosis, or vaccinated after diagnosis). Age was categorical to account for the nonlinear hospitalization and age association^30^ and different age distributions by race and ethnicity in our sample. Periods correspond roughly to peaks and troughs in the pandemic.^31,32^ While vaccines reduce COVID-19 severity, vaccination rates were uneven due to greater access barriers and vaccine hesitancy in some communities.^33^

### Statistical Analysis

We calculated summary statistics (mean [SD] and count [proportion]) for each key variable by hospitalization status and for neighborhood characteristics and hospitalization by race and ethnicity. We also calculated race and ethnicity–stratified means for each neighborhood characteristic by hospitalization status. Prior to conducting statistical comparisons by hospitalization status, we determined which racial and ethnic groups had sufficient sample size to detect a 5–percentile point HPI difference between hospitalized and nonhospitalized groups (α = .05; power, 0.8) (eTable 1 in Supplement 1). For groups with sufficient sample size (Black, Hispanic, and White), we used a 2-sample *t* test to compare mean neighborhood characteristics by hospitalization status.

We then constructed race and ethnicity–stratified logistic regression models for groups with sufficient sample size, estimating the odds of hospitalization with each of the following independent variables: (1) HPI, (2) Black segregation, (3) HPI plus Black segregation, (4) Hispanic segregation, (5) HPI plus Hispanic segregation, and (6) SVI, while adjusting for all control variables listed above. All models clustered SEs at the census tract level to account for within–census tract association. We considered 2-sided *P* < .05 as statistically significant. We compared models based on whether the independent variables of interest were associated with hospitalization and direction of associations. We conducted analyses using Stata, version 17.0 (StataCorp LLC).

## Results

Characteristics of the 19 495 veterans with COVID-19 included in the analysis (91.0% men and 9.0% women; 16.1% Black, 27.7% Hispanic, and 45.0% White; mean [SE] age, 57.21 [17.68] years) varied by hospitalization status (Table 1). Hospitalized veterans (n = 2893) were more likely to be Black (584 [20.2%] vs 2558 [15.4%]), older (mean [SD] age, 66.97 [14.9] vs 55.51 [17.58] years), and men (2755 [95.2%] vs 14983 [90.2%]) and to have low SES (681 [23.5%] vs 2425 [14.6%]) than nonhospitalized veterans. The most common Centers for Disease Control and Prevention risk factor was having overweight or obesity (15 612 [81.4%]). Compared with nonhospitalized veterans, hospitalized veterans lived in less healthy (lower HPI) and more vulnerable (higher SVI) neighborhoods with higher Black and Hispanic segregation.

**Table 1.** Sample Characteristics by Hospitalization Status Among Veterans Health Administration Users With COVID-19

Characteristic	Group with COVID-19^a^		*P* value^b^	All (N = 19 495)
	Hospitalized (n = 2893)	Not hospitalized (n = 16 602)		
Age, mean (SD), y	66.97 (14.90)	55.51 (17.58)	<.001	57.21 (17.68)
Sex				
Men	2755 (95.2)	14 983 (90.2)	<.001	17 738 (91.0)
Women	138 (4.8)	1619 (9.8)		1757 (9.0)
Race and ethnicity				
American Indian or Alaska Native	25 (0.9)	138 (0.8)	<.001	163 (0.8)
Asian	122 (4.2)	860 (5.2)		982 (5.0)
Black	584 (20.2)	2558 (15.4)		3142 (16.1)
Hispanic	661 (22.8)	4737 (28.5)		5398 (27.7)
Native Hawaiian or other Pacific Islander	55 (1.9)	287 (1.7)		342 (1.8)
White	1352 (46.7)	7418 (44.7)		8770 (45.0)
Unknown	94 (3.2)	604 (3.6)		698 (3.6)
SES (priority group)				
High	305 (10.5)	1987 (12.0)	<.001	2292 (11.8)
Low	681 (23.5)	2425 (14.6)		3106 (15.9)
Indeterminate	1905 (65.8)	12 177 (73.3)		14 082 (72.2)
Missing	2 (0.1)	13 (0.1)		15 (0.1)
CDC comorbidities				
Cancer	735 (25.4)	2228 (13.4)	<.001	2963 (15.2)
Chronic kidney disease	689 (23.8)	1448 (8.7)	<.001	2137 (11.0)
Chronic lung disease	1034 (35.7)	3704 (22.3)	<.001	4738 (24.3)
Dementia	304 (10.5)	644 (3.9)	<.001	948 (4.9)
Diabetes	1290 (44.6)	4131 (24.9)	<.001	5421 (27.8)
Heart disease	990 (34.2)	2425 (14.6)	<.001	3415 (17.5)
HIV	53 (1.8)	164 (1.0)	<.001	217 (1.1)
Liver disease	268 (9.3)	991 (6.0)	<.001	1259 (6.5)
Overweight or obesity^c^^,^^d^	2092 (72.6)	13 520 (83.0)	<.001	15 612 (81.4)
Pregnancy^d^	2 (0.1)	31 (0.2)	.15	33 (0.2)
Sickle cell anemia	5 (0.2)	18 (0.1)	.39	23 (0.1)
Ischemic stroke	253 (8.7)	568 (3.4)	<.001	821 (4.2)
Cerebrovascular disease	110 (3.8)	252 (1.5)	<.001	362 (1.9)
Drug and alcohol use disorder	616 (21.3)	4103 (24.7)	<.001	4719 (24.2)
Fully vaccinated^e^	314 (10.9)	1893 (11.4)	<.001	2207 (11.3)
COVID-19 periods				
Early (March 1 to May 31, 2020)	139 (4.8)	318 (1.9)	<.001	457 (2.3)
Summer surge (June 1 to July 31, 2020)	270 (9.3)	1303 (7.8)		1573 (8.1)
Fall drop (August 1 to October 31, 2020)	212 (7.3)	1001 (6.0)		1213 (6.2)
Winter surge (November 1, 2020, to February 28, 2021)	1468 (50.7)	9203 (55.4)		10671 (54.7)
Vaccine drop (March 1 to June 30, 2021	212 (7.3)	1192 (7.2)		1404 (7.2)
Delta surge (July 1 to October 31, 2021)	592 (20.5)	3585 (21.6)		4177 (21.4)
Neighborhood characteristics, mean (SD)				
HPI percentile^f^	41.78 (26.03)	44.85 (24.96)	<.001	44.38 (25.15)
Black segregation^g^	0.09 (0.13)	0.07 (0.10)	<.001	0.08 (0.11)
Hispanic segregation^g^	0.40 (0.23)	0.38 (0.23)	<.001	0.39 (0.23)
SVI percentile^h^	57.15 (26.37)	53.73 (25.90)	<.001	54.25 (26.00)

Abbreviations: CDC, Centers for Disease Control and Prevention; HPI, Healthy Places Index; SES, socioeconomic status; SVI, Social Vulnerability Index.

^a^ Unless otherwise indicated, data are expressed as No. (%) of veterans.

^b^ Compares hospitalized vs not hospitalized veterans.

^c^ Overweight or obesity was defined as a body mass index (calculated as weight in kilograms divided by height in meters squared) of 25 or greater.

^d^ Indicates at time of COVID-19 positive test result.

^e^ Received 2 doses of the messenger RNA vaccine or 1 dose of the adenovirus vaccine prior to COVID-19 positive test result.

^f^ Scores range from 1 to 100, with higher values representing neighborhoods with healthier characteristics.

^g^ Calculated as an isolation index ranging from 0 to 1, with higher values indicating greater segregation.

^h^ Scores range from 0 to 100, with higher values represent more socially vulnerable neighborhoods.

Neighborhood characteristics varied by race and ethnicity (Table 2). Asian veterans lived in the healthiest neighborhoods (mean [SD] HPI percentile, 52.28 [25.07]). Black and Hispanic veterans lived in the highest Black (mean [SD], 0.17 [0.18]) and Hispanic (mean [SD], 0.50 [0.24]) segregated neighborhoods, respectively. Black veterans lived in the most socially vulnerable neighborhoods (mean [SD] SVI, 60.59 [25.92]). The proportion of hospitalized veterans ranged from 122 (12.4%) among Asian veterans and 661 (12.2%) among Hispanic veterans to 584 (18.6%) among Black veterans.

**Table 2.** Neighborhood Characteristics and Hospitalization Stratified by Race and Ethnicity, Among Veterans Health Administration Users With COVID-19

	Racial or ethnic group^a^						*P* value	All (N = 19 495)
	American Indian or Alaska Native (n = 163)	Asian (n = 982)	Black (n = 3142)	Hispanic (n = 5398)	Native Hawaiian or other Pacific Islander (n = 342)	White (n = 8770)		
Neighborhood characteristics, mean (SD)								
HPI percentile^b^	41.04 (23.49)	52.28 (25.07)	38.83 (25.45)	39.61 (23.87)	45.70 (22.56)	48.42 (24.97)	<.001	44.39 (25.15)
Black segregation^c^	0.06 (0.06)	0.07 (0.08)	0.17 (0.18)	0.07 (0.08)	0.08 (0.10)	0.05 (0.07)	<.001	0.08 (0.11)
Hispanic segregation^c^	0.38 (0.23)	0.36 (0.20)	0.40 (0.21)	0.50 (0.24)	0.39 (0.20)	0.32 (0.21)	<.001	0.39 (0.23)
SVI percentile^d^	58.23 (24.99)	50.30 (25.51)	60.59 (25.92)	59.67 (25.26)	53.63 (23.37)	49.04 (25.51)	<.001	54.24 (26.00)
Hospitalization, No. (%)	25 (15.3)	122 (12.4)	584 (18.6)	661 (12.2)	55 (16.1)	1352 (15.4)	<.001	2893 (14.8)

Abbreviations: HPI, Healthy Places Index; SVI, Social Vulnerability Index.

^a^ Veterans with unknown race or ethnicity were included in the sample, but statistics for this group are not reported. Data are from March 1, 2020, to October 31, 2021.

^b^ Scores range from 0 to 100, with higher values representing neighborhoods with healthier characteristics.

^c^ Calculated as an isolation index ranging from 0 to 1, with higher values indicating greater segregation.

^d^ Scores range from 0 to 100, with higher values representing more socially vulnerable neighborhoods.

Table 3 presents mean neighborhood characteristics for those hospitalized and not hospitalized overall and by race and ethnicity. Black and White veterans who were hospitalized were more likely to live in less healthy, more socially vulnerable neighborhoods with higher Black and Hispanic segregation compared with their nonhospitalized counterparts. Hispanic veterans who were hospitalized also lived in less healthy and more socially vulnerable neighborhoods.

**Table 3.** Comparison of Neighborhood Characteristics by Hospitalization Status, Stratified by Race and Ethnicity Among Veterans Health Administration Users With COVID-19^a^

Neighborhood characteristic	Hospitalization status	American Indian or Alaska Native		Asian			Black		Native Hawaiian or other Pacific Islander		Hispanic		White		Total	
		Mean (SD)	*P* value	Mean (SD)	*P* value	Mean (SD)		*P* value	Mean (SD)	*P* value	Mean (SD)	*P* value	Mean (SD)	*P* value	Mean (SD)	*P* value
HPI percentile^b^	Hospitalized	48.47 (19.33)	NA^c^	52.18 (26.08)	NA^c^	34.84 (25.85)		<.001	42.27 (24.60)	NA^c^	37.15 (24.00)	.006	46.04 (26.01)	<.001	41.78 (26.03)	<.001
	Not hospitalized	39.76 (23.97)		52.35 (24.94)		39.76 (25.27)			46.36 (22.14)		39.97 (23.83)		48.85 (24.74)		44.85 (24.96)	
Black segregation^d^	Hospitalized	0.07 (0.08)	NA^c^	0.08 (0.10)	NA^c^	0.2 (0.20)		<.001	0.1 (0.09)	NA^c^	0.07 (0.08)	.13	0.06 (0.07)	<.001	0.09 (0.13)	<.001
	Not hospitalized	0.05 (0.06)		0.07 (0.08)		0.16 (0.17)			0.08 (0.10)		0.07 (0.08)		0.05 (0.06)		0.07 (0.10)	
Hispanic segregation^d^	Hospitalized	0.35 (0.21)	NA^c^	0.36 (0.21)	NA^c^	0.43 (0.22)		.003	0.43 (0.23)	NA^c^	0.51 (0.24)	.63	0.35 (0.21)	<.001	0.4 (0.23)	<.001
	Not hospitalized	0.38 (0.23)		0.36 (0.20)		0.4 (0.21)			0.38 (0.19)		0.5 (0.24)		0.31 (0.20)		0.38 (0.23)	
SVI percentile^e^	Hospitalized	53.09 (25.62)	NA^c^	53.09 (25.62)	NA^c^	64.51 (25.67)		<.001	55.66 (24.68)	NA^c^	62.06 (25.41)	.01	51.89 (26.11)	<.001	57.15 (26.37)	<.001
	Not hospitalized	59.44 (25.35)		49.92 (25.52)		59.66 (25.91)			53.35 (23.14)		59.33 (25.23)		48.52 (25.36)		53.73 (25.90)	

Abbreviations: HPI, Healthy Places Index; NA, not applicable; SVI, Social Vulnerability Index.

^a^ Veterans with unknown race or ethnicity were included in the sample, but statistics for this group are not reported. Data are from March 1, 2020, to October 31, 2021.

^b^ Scores range from 0 to 100, with higher values representing neighborhoods with healthier characteristics.

^c^ Statistical comparisons not conducted due to small sample size.

^d^ Calculated as an isolation index ranging from 0 to 1, with higher values indicating greater segregation.

^e^ Scores range from 0 to 100, with higher values represent more socially vulnerable neighborhoods.

### Regression Analysis 

Table 4 summarizes key regression results across all 6 models for each racial and ethnic group. Complete regression analysis results are found in eTables 2 to 4 in Supplement 1.

**Table 4.** Odds of Hospitalization Stratified by Race and Ethnicity Among Veterans Health Administration Users With COVID-19^a^

Neighborhood characteristic	Model 1: HPI		Model 2: Black segregation		Model 3: HPI plus Black segregation		Model 4: Hispanic segregation		Model 5: HPI plus Hispanic segregation		Model 6: SVI	
	OR (95% CI)	*P* value	OR (95% CI)	*P* value	OR (95% CI)	*P* value	OR (95% CI)	*P* value	OR (95% CI)	*P* value	OR (95% CI)	*P* value
**Black veterans**												
Lower HPI percentile	1.07 (1.03-1.12)	.001	NA	NA	1.06 (1.02-1.11)	.005	NA	NA	1.05 (1.00-1.11)	.06	NA	NA
Black segregation	NA	NA	1.95 (1.18-3.25)	.01	1.60 (0.93-2.77)	.09	NA	NA	NA	NA	NA	NA
Hispanic segregation	NA	NA	NA	NA	NA	NA	2.02 (1.25-3.25)	.004	1.50 (0.82-2.74)	.18	NA	NA
SVI	NA	NA	NA	NA	NA	NA	NA	NA	NA	NA	1.06 (1.02-1.10)	.006
**Hispanic veterans**												
Lower HPI percentile	1.03 (1.00-1.08)	.08	NA	NA	1.03 (0.99-1.07)	.16	NA	NA	1.04 (0.99-1.09)	.09	NA	NA
Black segregation	NA	NA	2.88 (1.03-8.07)	.04	2.90 (1.02-8.23)	.05	NA	NA	NA	NA	NA	NA
Hispanic segregation	NA	NA	NA	NA	NA	NA	1.17 (0.80-1.71)	.42	0.90 (0.55-1.45)	.65	NA	NA
SVI	NA	NA	NA	NA	NA	NA	NA	NA	NA	NA	1.03 (0.99-1.07)	.11
**White veterans**												
Lower HPI percentile	1.03 (1.00-1.06)	.03	NA	NA	1.02 (0.99-1.05)	.16	NA	NA	0.98 (0.95-1.02)	.23	NA	NA
Black segregation	NA	NA	5.36 (2.24-12.86)	<.001	4.42 (1.62-12.08)	.004	NA	NA	NA	NA	NA	NA
Hispanic segregation	NA	NA	NA	NA	NA	NA	2.46 (1.83-3.31)	<.001	2.81 (1.96-4.03)	<.001	NA	NA
SVI	NA	NA	NA	NA	NA	NA	NA	NA	NA	NA	1.04 (1.01-1.06)	.008

Abbreviations: HPI, Healthy Places Index; NA, not applicable; OR, odds ratio; SVI, Social Vulnerability Index.

^a^ Models are adjusted for sex, age, individual socioeconomic status, Centers for Disease Control and Prevention comorbidities, vaccination status prior to COVID-19 infection, and COVID-19 period. Results for American Indian or Alaska Native, Asian, and Native Hawaiian or other Pacific Islander veterans and veterans with unknown race or ethnicity are not shown due to small sample size. Odds ratios for HPI percentile and SVI are per 10–percentile point change. The HPI percentile has been reverse coded (lower HPI percentile) such that an OR less than 1.00 indicates lower risk of hospitalization and OR of greater than 1.00 indicates greater risk of hospitalization for all measures.

#### Black Veterans

Lower HPI (OR, 1.07 [95% CI, 1.03-1.12]), higher Black segregation (OR, 1.95 [95% CI, 1.18-3.25]), higher Hispanic segregation (OR, 2.02 [95% CI, 1.25-3.25]), and higher SVI (OR, 1.06 [95% CI, 1.02-1.10]) were separately associated with greater odds of hospitalization among Black veterans with COVID-19. In the model with both HPI and Black segregation, HPI remained associated with hospitalization (OR, 1.06 [95% CI, 1.02-1.11]); Black segregation was no longer associated with hospitalization (OR, 1.60 [95% CI, 0.93-2.77]). When accounting for both HPI and Hispanic segregation, HPI (OR, 1.05 [95% CI, 1.00-1.11] and Hispanic segregation (OR, 1.50 [95% CI, 0.82, 2.74]) were no longer associated with hospitalization.

#### Hispanic Veterans

The HPI’s association with hospitalization did not reach statistical significance among Hispanic veterans with COVID-19–positive test results when modeled alone (OR, 1.03 [95% CI, 1.00-1.08) and with Hispanic segregation (OR, 1.04 [95% CI, 0.99-1.09]). Black segregation was associated with greater hospitalization alone (OR, 2.88 [95% CI, 1.03-8.07]) and with HPI adjustment (OR, 2.90 [95% CI, 1.02-8.23]). Hispanic segregation and SVI were not associated with hospitalization.

#### White Veterans

Lower HPI (OR, 1.03 [95% CI, 1.00-1.06]), higher Black segregation (OR, 5.36 [95% CI, 2.24-12.86]), higher Hispanic segregation (OR, 2.46 [95% CI, 1.83-3.31]), and higher SVI (OR, 1.04 [95% CI, 1.01-1.06]) were separately associated with greater hospitalization among White veterans with COVID-19. When both the HPI and Black segregation were in the same model, Black segregation remained associated with hospitalization (OR, 4.42 [95% CI, 1.62-12.08]), while HPI was not (OR, 1.02 [95% CI, 0.99-1.05]). In the model with both Hispanic segregation and HPI, Hispanic segregation (OR, 2.81 [95% CI, 1.96-4.03]), but not HPI (OR, 0.98 [95% CI, 0.95-1.02]), was associated with hospitalization.

## Discussion

In this cohort study, we found that among veterans with COVID-19, living in neighborhoods in California with lower HPI was associated with greater COVID-19–related hospitalization for Black and White veterans after adjusting for individual-level confounders and period. This association did not change for Black veterans when we also accounted for Black segregation, but among White veterans, living in lower-HPI neighborhoods was no longer associated with increased hospitalization when we accounted for Black or Hispanic segregation. For Hispanic veterans, lower HPI was not associated with greater hospitalization alone and when we accounted for Hispanic segregation. Residing in Black segregated neighborhoods was associated with greater hospitalization for Hispanic and White veterans with COVID-19, and residing in Hispanic segregated neighborhoods was associated with greater hospitalization for White veterans.

There are multiple ways to measure neighborhood deprivation. Indices capturing multiple facets of neighborhood deprivation are particularly valuable for identifying high-risk neighborhoods as they capture complexities underlying neighborhood-level vulnerability. Neighborhood risk for severe COVID-19 likely results from the confluence of multiple neighborhood attributes rather than any single attribute. It is important to ensure that single indices aiming to capture the multidimensional nature of neighborhood deprivation also account for the social and economic impacts of segregation. Had we found that HPI underestimated the odds of hospitalization among racial and ethnic minority populations with COVID-19 after accounting for segregation, this could have potential implications for inequities in resource allocation and programming. However, our findings suggest that although HPI does not include racial and ethnic segregation, it adequately captures risk specifically for COVID-19–related hospitalization. Developers of HPI were aware of this potential limitation from California’s 1996 ballot proposition 209 and thus designed HPI to incorporate numerous other measures that potentially capture the processes that result from segregation (eg, park access, clean environment, and retail availability). Moreover, our findings of HPI and COVID-19–related hospitalization are consistent with the CDPH’s own analyses of HPI and COVID-19 mortality by race and ethnicity.^18,19^ Our HPI findings are also comparable with our findings with SVI, which is commonly used to identify communities vulnerable to adverse health outcomes^34,35^ and hazardous events,^36^ including the COVID-19 pandemic,^37,38^ which strengthens our confidence in HPI’s ability to estimate outcomes.

We found that living in Black segregated neighborhoods was associated with hospitalization for Hispanic and White veterans with COVID-19 after accounting for HPI. We were surprised that the strongest association with Black segregation was among White veterans and the weakest association was among Black veterans. There are several potential reasons why segregation may be more strongly associated with hospitalization among White and Hispanic veterans than among Black veterans. First, Black segregated neighborhoods in California include lower- and middle-income and recently gentrifying communities.^39^ It is possible that in our study, while Black veterans lived in both middle- and lower-income Black segregated neighborhoods, White and Hispanic veterans living in Black segregated neighborhoods predominately resided in neighborhoods with more socioeconomic deprivation.^39,40^ Additionally, these Hispanic and White veterans may be those with the fewest resources, such as those who are formerly homeless and have few housing options available through the VA’s supportive housing program.^41^ Furthermore, it is possible that while living in Black segregated neighborhoods provides both benefits and challenges to Black veterans (eg, closer social ties^42,43^ and efforts to counter medical distrust and disinformation about COVID-19), residents of other races and ethnicities living in these neighborhoods did not experience these benefits.^44^

Associations between neighborhood socioeconomic deprivation and COVID-19 are complex and vary by race and ethnicity. Few studies have considered these differences by race and ethnicity,^45,46^ and these studies have excluded smaller racial and ethnic groups. However, the CDPH’s race and place analyses, which found excess mortality among Native Hawaiian or other Pacific Islander Californians after accounting for HPI,^18^ underscore the need to examine disparities specifically among smaller racial and ethnic groups. While our study sample was underpowered to examine adjusted associations for American Indian or Alaska Native, Asian, and Native Hawaiian or other Pacific Islander groups, unadjusted descriptive statistics of neighborhood characteristics suggest potential differences in COVID-19 severity among some of these racial and ethnic groups. We chose not to conduct statistical comparisons in groups underpowered to detect differences, because statistical testing could lead us to erroneously conclude no evidence existed of a difference between hospitalized and nonhospitalized groups when, in fact, we were underpowered. This could lead to potentially deleterious conclusions for these groups. Instead, unadjusted means in our analysis posit associations that warrant further investigations, including between HPI and hospitalization among American Indian or Alaska Native and Native Hawaiian or other Pacific Islander individuals, and between Hispanic segregation and hospitalization among Native Hawaiian or other Pacific Islander individuals. Future research can focus on these groups, including ways to augment sample size, and how Hispanic or Black segregation is associated with their health outcomes.

### Strengths and Limitations

A strength of this study is that we examined a multiethnic sample with well-characterized demographic and health characteristics for these race and ethnicity–stratified analyses. Study limitations include limited generalizability to nonveterans, populations beyond California, all VHA users including veterans without COVID-19, cross-sectional data preventing causal inferences, possible misclassification of hospitalization to include incidental COVID-19 hospitalizations, and insufficient sample size to run statistical analyses for some racial and ethnic groups that have been disproportionately affected by COVID-19 (eg, American Indian or Alaska Native, Native Hawaiian or other Pacific Islander groups).

## Conclusions

In this cohort study of veterans with COVID-19 residing in California, associations between HPI and hospitalization varied by race and ethnicity. Our findings have implications for using HPI and other composite neighborhood deprivation indices that do not explicitly account for racial and ethnic segregation. This potential limitation of HPI has been acknowledged, and the index incorporates several measures potentially capturing underlying processes through which segregation can affect the health of racial and ethnic minority populations. We found that HPI captures neighborhood-level risk for COVID-19–related hospitalization among Black, Hispanic, and White veterans with COVID-19, but also that accounting for segregation provided additional information for some racial and ethnic groups. Our exploratory research among smaller racial and ethnic groups is a call to action for additional data collection and analyses for these groups. To understand the association of place and health, it is important to ensure that composite measures, including HPI, accurately account for structural racism’s effect on neighborhood deprivation for different racial and ethnic groups.

## Notes

Supplement 1. **eMethods.** Description of the Creation of the Isolation Index **eTable 1.** Comparison of Required vs Actual Sample Size for Racial and Ethnic Groups Excluded From Statistical Analyses Due to Insufficient Sample Size **eTable 2.** Full Regression Results for Non-Hispanic Black Veterans With Positive Test Results for COVID-19 **eTable 3.** Full Regression Results for Hispanic Veterans With Positive Test Results for COVID-19 **eTable 4.** Full Regression Results for Non-Hispanic White Veterans With Positive Test Results for COVID-19

Supplement 2.
Data Sharing Statement
