# Supplementary material for: Inclusion of Race and Ethnicity With Neighborhood Socioeconomic Deprivation When Assessing COVID-19 Hospitalization Risk Among California Veterans Health Administration Users
Source: JAMA Netw Open. 2023 Mar 3;6(3):e231471. doi: 10.1001/jamanetworkopen.2023.1471 (PMC9984969; doi:10.1001/jamanetworkopen.2023.1471)
Supplement: Supplement 1. — eMethods. Description of the Creation of the Isolation Index eTable 1. Comparison of Required vs Actual Sample Size for Racial and Ethnic Groups Excluded From Statistical Analyses Due to Insufficient Sample Size eTable 2. Full Regression Results for Non-Hispanic Black Veterans With Positive Test Results for COVID-19 eTable 3. Full Regression Results for Hispanic Veterans With Positive Test Results for COVID-19 eTable 4. Full Regression Results for Non-Hispanic White Veterans With Positive Test Results for COVID-19 [file jamanetwopen-e231471-s001.pdf]

Supplementary Online Content

Wong MS, Brown AF, Washington DL. Inclusion of race and ethnicity with neighborhood socioeconomic deprivation when assessing COVID-19 hospitalization risk among California Veterans Health Administration users. *JAMA Netw Open*. 2023;6(3):e231471. doi:10.1001/jamanetworkopen.2023.1471

eMethods. Description of the Creation of the Isolation Index
eTable 1. Comparison of Required vs Actual Sample Size for Racial and Ethnic Groups Excluded From Statistical Analyses Due to Insufficient Sample Size
eTable 2. Full Regression Results for Non-Hispanic Black Veterans With Positive Test Results for COVID-19
eTable 3. Full Regression Results for Hispanic Veterans With Positive Test Results for COVID-19
eTable 4. Full Regression Results for Non-Hispanic White Veterans With Positive Test Results for COVID-19

This supplementary material has been provided by the authors to give readers additional information about their work.

© 2023 Wong MS et al. *JAMA Network Open*.

eMethods. Description of the Creation of the Isolation Index

Isolation Index. This measure captures the probability that a Black or Hispanic person exposed to another Black or Hispanic person within a census tract. We calculated the isolation index with the following formula from Massey and Denton (1988)[1]:

$$\sum_{i=1}^{n}\left[\left(\frac{x_i}{X}\right)\left(\frac{x_i}{t_i}\right)\right]$$

Where n = the number of census block groups ("areas") in the census tract ("metropolitan area") ranked smallest to largest by land area (Massey and Denton created the isolation index at the county level where the "areas" represented by n were census tracts and the "metropolitan area" was the county. However, as we note, we calculated the isolation index at a smaller geographic unit of analysis, where the "areas" were census block groups and the "metropolitan area" were census tracts

x_i = the racial or ethnic segregated group (i.e., Black or Hispanic) population of census block group ("area") i

X = sum of all x_i (i.e., the total segregated group population in census tract)

t_i = total population of census block group ("area") i

Reference:

1. Massey DS, Denton NA. The Dimensions of Residential Segregation. *Social Forces*. 1988;67(2):281-315.

© 2023 Wong MS et al. *JAMA Network Open*.

eTable 1. Comparison of Required vs Actual Sample Size for Racial and Ethnic Groups Excluded From Statistical Analyses Due to Insufficient Sample Size

Racial and ethnic group	Required sample size	Actual sample size
AI/AN	1083	163
Asian	1944	989
NH/OPI	1420	343

Note: AI/AN denotes American Indian/Alaska Native, NH/OPI denotes Native Hawaiian/Other Pacific Islander. We calculate sample sizes required to detect a 5 percentage point difference in HPI between the hospitalized and not hospitalized groups (i.e., effect size), assuming alpha = 0.05, power = 0.8, and the ratio of the proportion hospitalized and not hospitalized for each racial and ethnic group

© 2023 Wong MS et al. *JAMA Network Open*.

eTable 2. Full Regression Results for Non-Hispanic Black Veterans With Positive Test Results for COVID-19

	Model 1: HPI		Model 2: Black Segregation		Model 3: HPI + Black Segregation		Model 4: Hispanic Segregation		Model 5: HPI + Hispanic Segregation		Model 6:SVI	
	OR (95%CI)	P-val	OR (95%CI)	P-val	OR (95%CI)	P-val	OR (95%CI)	P-val	OR (95%CI)	P-val	OR (95%CI)	P-val
Lower HPI Percentile	1.07 (1.03-1.12)	0.001			1.06 (1.02-1.11)	0.005			1.05 (0.997-1.11)	0.06		
Black Segregation			1.95 (1.18-3.25)	0.01	1.60 (0.93-2.77)	0.09						
Hispanic Segregation							2.02 (1.25-3.25)	0.004	1.50 (0.82-2.74)	0.18		
SVI						.				.	1.06 (1.02-1.10)	0.006
Female	0.88 (0.58-1.33)	0.54	0.85 (0.57-1.28)	0.45	0.88 (0.58-1.33)	0.54	0.85 (0.56-1.29)	0.45	0.88 (0.58-1.33)	0.55	0.86 (0.57-1.30)	0.48
Age Category												
<60 years	Ref		Ref		Ref		Ref		Ref		Ref	
60-64 yrs	1.54 (1.10-2.16)	0.01	1.60 (1.15-2.24)	0.006	1.54 (1.10-2.16)	0.01	1.60 (1.15-2.23)	0.006	1.54 (1.10-2.16)	0.01	1.56 (1.12-2.18)	0.008
65-69 yrs	1.81 (1.30-2.52)	<0.001	1.83 (1.32-2.54)	<0.001	1.79 (1.28-2.50)	0.001	1.86 (1.34-2.58)	<0.001	1.81 (1.30-2.53)	<0.001	1.82 (1.32-2.53)	<0.001
70-74 yrs	2.87 (2.06-4.01)	<0.001	2.77 (1.99-3.85)	<0.001	2.85 (2.04-3.98)	<0.001	2.87 (2.07-3.98)	<0.001	2.89 (2.07-4.03)	<0.001	2.81 (2.02-3.91)	<0.001
75-79 yrs	2.36 (1.60-3.49)	<0.001	2.27 (1.54-3.35)	<0.001	2.30 (1.56-3.41)	<0.001	2.36 (1.60-3.49)	<0.001	2.37 (1.61-3.51)	<0.001	2.31 (1.57-3.40)	<0.001
80+ yrs	3.35 (2.27-4.94)	<0.001	3.33 (2.27-4.87)	<0.001	3.29 (2.23-4.86)	<0.001	3.45 (2.36-5.05)	<0.001	3.35 (2.28-4.94)	<0.001	3.40 (2.32-4.98)	<0.001
SES												
High SES	Ref		Ref		Ref		Ref		Ref		Ref	
Low SES	1.34 (0.88-2.04)	0.18	1.41 (0.93-2.13)	0.10	1.36 (0.89-2.07)	0.16	1.37 (0.91-2.07)	0.13	1.34 (0.88-2.03)	0.18	1.35 (0.90-2.03)	0.15
Indeterminate	1.43 (0.98-2.09)	0.06	1.45 (1.00-2.11)	0.05	1.46 (1.00-2.14)	0.05	1.41 (0.97-2.04)	0.07	1.43 (0.98-2.09)	0.06	1.41 (0.97-2.04)	0.07
Comorbidities												
Cancer	1.26 (0.99-1.62)	0.06	1.23 (0.96-1.57)	0.10	1.23 (0.96-1.59)	0.10	1.27 (0.99-1.61)	0.06	1.27 (0.99-1.62)	0.06	1.25 (0.98-1.60)	0.07
Chronic Kidney Disease	1.71 (1.33-2.19)	<0.001	1.69 (1.32-2.16)	<0.001	1.71 (1.33-2.20)	<0.001	1.67 (1.30-2.14)	<0.001	1.70 (1.33-2.19)	<0.001	1.67 (1.31-2.15)	<0.001
Chronic Lung Disease	1.16 (0.92-1.45)	0.20	1.18 (0.94-1.47)	0.15	1.17 (0.93-1.46)	0.18	1.16 (0.92-1.45)	0.21	1.16 (0.92-1.45)	0.21	1.16 (0.93-1.45)	0.19
Dementia	0.86 (0.57-1.29)	0.46	0.78 (0.51-1.17)	0.23	0.87 (0.58-1.31)	0.50	0.77 (0.51-1.16)	0.22	0.85 (0.57-1.28)	0.44	0.78 (0.52-1.19)	0.25
Diabetes	1.60 (1.29-1.98)	<0.001	1.56 (1.25-1.94)	<0.001	1.60 (1.29-1.98)	<0.001	1.57 (1.26-1.95)	<0.001	1.61 (1.30-1.99)	<0.001	1.56 (1.25-1.94)	<0.001
Heart Disease	1.41 (1.09-1.81)	0.008	1.43 (1.11-1.83)	0.005	1.40 (1.09-1.80)	0.009	1.43 (1.12-1.83)	0.005	1.40 (1.09-1.81)	0.008	1.43 (1.11-1.82)	0.005
HIV	1.27 (0.66-2.47)	0.48	1.23 (0.65-2.34)	0.53	1.28 (0.66-2.46)	0.47	1.26 (0.65-2.43)	0.50	1.28 (0.66-2.50)	0.46	1.24 (0.64-2.39)	0.52
Liver Disease	1.07 (0.67-1.69)	0.78	1.09 (0.70-1.70)	0.71	1.07 (0.68-1.71)	0.76	1.05 (0.67-1.63)	0.83	1.05 (0.67-1.66)	0.83	1.07 (0.69-1.67)	0.77

© 2023 Wong MS et al. *JAMA Network Open*.

Overweight or Obese[a]	0.64 (0.50-0.81)	<0.001	0.64 (0.50-0.81)	<0.001	0.64 (0.50-0.82)	<0.001	0.63 (0.49-0.80)	<0.001	0.63 (0.49-0.81)	<0.001	0.64 (0.50-0.81)	<0.001
Pregnancy[a]	--		--		--		--		--		--	
Sickle Cell	0.60 (0.13-2.82)	0.52	0.59 (0.12-2.80)	0.50	0.59 (0.12-2.87)	0.52	0.60 (0.13-2.68)	0.50	0.60 (0.13-2.80)	0.52	0.61 (0.14-2.71)	0.51
Ischemic Stroke	1.10 (0.73-1.64)	0.65	1.10 (0.73-1.64)	0.65	1.10 (0.73-1.64)	0.65	1.06 (0.71-1.59)	0.78	1.09 (0.73-1.62)	0.69	1.08 (0.72-1.61)	0.72
Cerebrovascular Disease	0.89 (0.53-1.50)	0.66	0.94 (0.55-1.61)	0.81	0.87 (0.52-1.46)	0.59	0.98 (0.57-1.67)	0.94	0.89 (0.53-1.50)	0.67	0.97 (0.57-1.65)	0.91
Alcohol or Drug Disorder	1.03 (0.80-1.32)	0.84	1.02 (0.80-1.30)	0.89	1.02 (0.79-1.30)	0.90	1.04 (0.81-1.33)	0.76	1.03 (0.80-1.32)	0.82	1.02 (0.80-1.30)	0.88
Vaccine status before COVID-19 Infection[b]												
Not Fully	Ref		Ref		Ref		Ref		Ref		Ref	
Fully vaccinated[c]	0.38 (0.25-0.57)	<0.001	0.38 (0.25-0.58)	<0.001	0.38 (0.25-0.57)	<0.001	0.38 (0.25-0.57)	<0.001	0.38 (0.25-0.57)	<0.001	0.38 (0.25-0.58)	<0.001
COVID-19 Time Period												
Early	Ref		Ref		Ref		Ref		Ref		Ref	
Summer Surge	0.50 (0.29-0.86)	0.01	0.54 (0.31-0.93)	0.03	0.52 (0.30-0.89)	0.02	0.51 (0.30-0.88)	0.02	0.50 (0.29-0.86)	0.01	0.52 (0.30-0.89)	0.02
Fall Drop	0.54 (0.31-0.96)	0.04	0.59 (0.34-1.04)	0.07	0.56 (0.32-1.00)	0.05	0.58 (0.33-1.02)	0.06	0.56 (0.31-0.98)	0.04	0.58 (0.33-1.01)	0.05
Winter Surge	0.41 (0.26-0.63)	<0.001	0.44 (0.28-0.68)	<0.001	0.42 (0.27-0.66)	<0.001	0.42 (0.27-0.65)	<0.001	0.41 (0.26-0.64)	<0.001	0.42 (0.27-0.65)	<0.001
Vaccine Drop	0.52 (0.29-0.94)	0.03	0.57 (0.32-1.01)	0.06	0.54 (0.30-0.98)	0.04	0.55 (0.31-0.97)	0.04	0.53 (0.29-0.96)	0.04	0.54 (0.30-0.96)	0.04
Delta Surge	0.73 (0.44-1.22)	0.23	0.75 (0.45-1.24)	0.26	0.76 (0.45-1.26)	0.29	0.74 (0.45-1.23)	0.25	0.75 (0.45-1.25)	0.27	0.72 (0.43-1.19)	0.20

Notes: a. at time of COVID-19 positive test b. individual who got vaccinated after getting COVID-19 were included as a separate group, but results are not shown; c. fully vaccinated = 2 mRNA vaccines or 1 adenovirus vaccine; -- not calculated due to empty cells; ORs for HPI percentile and SVI are per 10 percentile change. HPI percentile has been reverse coded (indicated with label lower HPI percentile) such that OR<1 indicates lower risk of hospitalization and OR>1 indicates greater risk of hospitalization; COVID-19 Time periods correspond to the following dates: Early (March - May 2020); Summer surge (June - July 2020); Fall drop (Aug - Oct 2020); winter surge (Nov 2020 - Feb 2021); Vaccine drop (March - June 2021); Delta Surge (July - Oct 2021)

© 2023 Wong MS et al. *JAMA Network Open*.

eTable 3. Full Regression Results for Hispanic Veterans With Positive Test Results for COVID-19

	Model 1: HPI		Model 2: Black Segregation		Model 3: HPI + Black Segregation		Model 4: Hispanic Segregation		Model 5: HPI + Hispanic Segregation		Model 6:SVI	
	OR (95%CI)	P-val	OR (95%CI)	P-val	OR (95%CI)	P-val	OR (95%CI)	P-val	OR (95%CI)	P-val	OR (95%CI)	P-val
Lower HPI Percentile	1.03 (0.996-1.08)	0.08			1.03 (0.99-1.07)	0.16			1.04 (0.99-1.09)	0.09		
Black Segregation			2.88 (1.03-8.07)	0.04	2.90 (1.02-8.23)	0.05						
Hispanic Segregation							1.17 (0.80-1.71)	0.42	0.90 (0.55-1.45)	0.65		
SVI											1.03 (0.99-1.07)	0.11
Female	0.82 (0.55-1.22)	0.32	0.83 (0.56-1.23)	0.36	0.82 (0.55-1.22)	0.33	0.83 (0.56-1.23)	0.36	0.82 (0.55-1.21)	0.32	0.83 (0.56-1.23)	0.36
Age Category												
<60 years	Ref		Ref		Ref		Ref		Ref		Ref	
60-64 yrs	1.63 (1.17-2.27)	0.004	1.59 (1.14-2.21)	0.006	1.64 (1.18-2.28)	0.003	1.58 (1.14-2.20)	0.006	1.63 (1.17-2.26)	0.004	1.57 (1.13-2.19)	0.007
65-69 yrs	1.77 (1.26-2.48)	0.001	1.76 (1.26-2.47)	0.001	1.78 (1.28-2.50)	0.001	1.76 (1.26-2.46)	0.001	1.77 (1.26-2.47)	0.001	1.75 (1.25-2.44)	0.001
70-74 yrs	2.01 (1.51-2.67)	<0.001	1.97 (1.48-2.63)	<0.001	2.02 (1.52-2.70)	<0.001	1.97 (1.48-2.62)	<0.001	2.00 (1.50-2.66)	<0.001	1.96 (1.48-2.61)	<0.001
75-79 yrs	2.12 (1.44-3.13)	<0.001	2.15 (1.47-3.16)	<0.001	2.15 (1.46-3.17)	<0.001	2.14 (1.46-3.14)	<0.001	2.12 (1.44-3.13)	<0.001	2.13 (1.45-3.12)	<0.001
80+ yrs	2.82 (1.86-4.29)	<0.001	2.78 (1.83-4.22)	<0.001	2.84 (1.87-4.32)	<0.001	2.77 (1.83-4.21)	<0.001	2.81 (1.85-4.27)	<0.001	2.75 (1.82-4.17)	<0.001
SES												
High SES	Ref		Ref		Ref		Ref		Ref		Ref	
Low SES	1.56 (1.12-2.17)	0.009	1.57 (1.12-2.19)	0.008	1.54 (1.10-2.15)	0.01	1.59 (1.14-2.21)	0.006	1.55 (1.11-2.17)	0.009	1.56 (1.12-2.18)	0.008
Indeterminate	0.97 (0.72-1.31)	0.86	0.98 (0.73-1.31)	0.87	0.97 (0.72-1.31)	0.862	0.98 (0.73-1.31)	0.88	0.97 (0.72-1.31)	0.85	0.97 (0.73-1.31)	0.86
Comorbidities												
Cancer	1.49 (1.16-1.92)	0.002	1.52 (1.18-1.96)	0.001	1.49 (1.15-1.92)	0.002	1.53 (1.19-1.96)	0.001	1.49 (1.15-1.92)	0.002	1.52 (1.18-1.96)	0.001
Chronic Kidney Disease	2.16 (1.63-2.88)	<0.001	2.16 (1.62-2.86)	<0.001	2.16 (1.63-2.87)	<0.001	2.16 (1.63-2.87)	<0.001	2.16 (1.63-2.88)	<0.001	2.16 (1.63-2.87)	<0.001
Chronic Lung Disease	1.17 (0.94-1.45)	0.17	1.16 (0.93-1.44)	0.18	1.17 (0.94-1.46)	0.16	1.16 (0.93-1.45)	0.18	1.17 (0.94-1.45)	0.17	1.16 (0.93-1.44)	0.19
Dementia	1.16 (0.75-1.80)	0.49	1.16 (0.75-1.78)	0.51	1.15 (0.75-1.78)	0.52	1.17 (0.76-1.79)	0.47	1.17 (0.76-1.80)	0.49	1.17 (0.76-1.79)	0.47
Diabetes	1.61 (1.31-1.99)	<0.001	1.62 (1.31-1.99)	<0.001	1.62 (1.32-2.00)	<0.001	1.60 (1.31-1.97)	<0.001	1.61 (1.31-1.99)	<0.001	1.60 (1.30-1.97)	<0.001
Heart Disease	1.55 (1.19-2.03)	0.001	1.57 (1.20-2.04)	0.001	1.56 (1.19-2.03)	0.001	1.56 (1.20-2.04)	0.001	1.56 (1.19-2.03)	0.001	1.56 (1.20-2.04)	0.001
HIV	2.10 (0.95-4.65)	0.07	2.21 (1.05-4.64)	0.04	2.11 (0.95-4.65)	0.07	2.23 (1.06-4.69)	0.04	2.11 (0.95-4.66)	0.07	2.19 (1.04-4.62)	0.04

© 2023 Wong MS et al. *JAMA Network Open*.

Liver Disease	1.32 (0.98-1.78)	0.07	1.32 (0.99-1.78)	0.06	1.33 (0.99-1.79)	0.06	1.32 (0.98-1.77)	0.07	1.32 (0.98-1.78)	0.07	1.31 (0.98-1.77)	0.07
Overweight or Obese[a]	0.77 (0.60-1.00)	0.05	0.77 (0.60-0.99)	0.04	0.77 (0.59-0.99)	0.04	0.77 (0.60-1.00)	0.05	0.77 (0.60-1.00)	0.05	0.77 (0.60-1.00)	0.05
Pregnancy[a]	1.92 (0.40-9.09)	0.41	1.87 (0.40-8.69)	0.42	1.94 (0.41-9.27)	0.40	1.84 (0.40-8.54)	0.44	1.92 (0.41-9.11)	0.41	1.84 (0.39-8.74)	0.44
Sickle Cell	4.31 (0.03-732.89)	0.58	3.91 (0.02-805.89)	0.62	4.22 (0.02-831.10)	0.59	3.95 (0.03-620.48)	0.59	4.36 (0.02-805.65)	0.58	4.11 (0.02-719.74)	0.59
Ischemic Stroke	1.02 (0.63-1.64)	0.95	1.01 (0.62-1.62)	0.98	1.02 (0.63-1.64)	0.95	1.01 (0.62-1.62)	0.98	1.01 (0.63-1.64)	0.95	1.01 (0.63-1.63)	0.96
Cerebrovascular Disease	1.16 (0.56-2.40)	0.69	1.19 (0.58-2.44)	0.63	1.14 (0.55-2.39)	0.72	1.21 (0.59-2.46)	0.60	1.16 (0.56-2.40)	0.69	1.20 (0.59-2.44)	0.62
Alcohol or Drug Disorder	1.01 (0.82-1.24)	0.93	1.01 (0.82-1.24)	0.93	1.02 (0.83-1.25)	0.89	1.00 (0.81-1.23)	0.99	1.01 (0.82-1.24)	0.91	1.00 (0.81-1.23)	0.99
Vaccine status before COVID-19 Infection[b]												
Not Fully	Ref		Ref		Ref		Ref		Ref		Ref	
Fully vaccinated[c]	0.50 (0.34-0.73)	<0.001	0.49 (0.33-0.71)	<0.001	0.49 (0.34-0.73)	<0.001	0.49 (0.33-0.72)	<0.001	0.50 (0.34-0.74)	<0.001	0.49 (0.33-0.72)	<0.001
COVID-19 Time Period												
Early	Ref		Ref		Ref		Ref		Ref		Ref	
Summer Surge	0.78 (0.42-1.43)	0.42	0.81 (0.44-1.48)	0.49	0.79 (0.43-1.44)	0.44	0.80 (0.44-1.46)	0.46	0.77 (0.42-1.42)	0.41	0.79 (0.43-1.44)	0.44
Fall Drop	0.69 (0.36-1.32)	0.26	0.71 (0.38-1.36)	0.31	0.69 (0.36-1.33)	0.27	0.71 (0.37-1.35)	0.29	0.69 (0.36-1.31)	0.26	0.70 (0.37-1.34)	0.28
Winter Surge	0.54 (0.31-0.96)	0.04	0.56 (0.32-0.99)	0.05	0.55 (0.31-0.97)	0.04	0.56 (0.32-0.98)	0.04	0.54 (0.31-0.96)	0.04	0.55 (0.31-0.98)	0.04
Vaccine Drop	0.93 (0.49-1.79)	0.84	0.97 (0.51-1.84)	0.92	0.95 (0.50-1.82)	0.88	0.96 (0.50-1.82)	0.89	0.93 (0.49-1.78)	0.83	0.95 (0.50-1.81)	0.87
Delta Surge	0.89 (0.47-1.66)	0.71	0.92 (0.50-1.72)	0.80	0.90 (0.48-1.68)	0.73	0.92 (0.49-1.71)	0.79	0.88 (0.47-1.66)	0.70	0.92 (0.49-1.71)	0.78

Notes: a. at time of COVID-19 positive test b. individual who got vaccinated after getting COVID-19 were included as a separate group, but results are not shown; c. fully vaccinated = 2 mRNA vaccines or 1 adenovirus vaccine; ORs for HPI percentile and SVI are per 10 percentile change. HPI percentile reverse coded (indicated with label lower HPI percentile) such that OR<1=lower risk of hospitalization and OR>1 = greater risk of hospitalization; COVID-19 Time periods correspond to the following dates: Early (March - May 2020); Summer surge (June - July 2020); Fall drop (Aug - Oct 2020); winter surge (Nov 2020 - Feb 2021); Vaccine drop (March - June 2021); Delta Surge (July - Oct 2021)

© 2023 Wong MS et al. *JAMA Network Open*.

eTable 4. Full Regression Results for Non-Hispanic White Veterans With Positive Test Results for COVID-19

	Model 1: HPI		Model 2: Black Segregation		Model 3: HPI + Black Segregation		Model 4: Hispanic Segregation		Model 5: HPI + Hispanic Segregation		Model 6:SVI	
	OR (95%CI)	P-val	OR (95%CI)	P-val	OR (95%CI)	P-val	OR (95%CI)	P-val	OR (95%CI)	P-val	OR (95%CI)	P-val
Lower HPI Percentile	1.03 (1.003-1.06)	0.03			1.02 (0.99-1.05)	0.16			0.98 (0.95-1.02)	0.23		
Black Segregation			5.36 (2.24-12.86)	<0.001	4.42 (1.62-12.08)	0.004						
Hispanic Segregation							2.46 (1.83-3.31)	<0.001	2.81 (1.96-4.03)	<0.001		
SVI											1.04 (1.01-1.06)	0.008
Female	0.76 (0.55-1.04)	0.09	0.76 (0.56-1.04)	0.09	0.76 (0.55-1.04)	0.09	0.76 (0.56-1.05)	0.10	0.77 (0.56-1.05)	0.10	0.76 (0.55-1.04)	0.09
Age Category												
<60 years	Ref		Ref		Ref		Ref		Ref		Ref	
60-64 yrs	1.61 (1.23-2.10)	0.001	1.64 (1.26-2.14)	<0.001	1.62 (1.23-2.12)	<0.001	1.62 (1.24-2.11)	<0.001	1.62 (1.23-2.12)	<0.001	1.61 (1.24-2.10)	<0.001
65-69 yrs	1.97 (1.53-2.53)	<0.001	2.02 (1.58-2.59)	<0.001	2.00 (1.56-2.57)	<0.001	1.99 (1.55-2.55)	<0.001	2.00 (1.56-2.57)	<0.001	1.97 (1.54-2.53)	<0.001
70-74 yrs	2.36 (1.92-2.90)	<0.001	2.38 (1.94-2.93)	<0.001	2.41 (1.96-2.97)	<0.001	2.35 (1.92-2.89)	<0.001	2.41 (1.96-2.97)	<0.001	2.32 (1.89-2.85)	<0.001
75-79 yrs	2.42 (1.89-3.08)	<0.001	2.42 (1.90-3.08)	<0.001	2.47 (1.93-3.15)	<0.001	2.42 (1.90-3.09)	<0.001	2.49 (1.95-3.19)	<0.001	2.36 (1.86-3.01)	<0.001
80+ yrs	2.59 (2.02-3.33)	<0.001	2.64 (2.06-3.38)	<0.001	2.65 (2.06-3.40)	<0.001	2.67 (2.09-3.42)	<0.001	2.71 (2.11-3.49)	<0.001	2.58 (2.01-3.30)	<0.001
SES												
High SES	Ref		Ref		Ref		Ref		Ref		Ref	
Low SES	1.52 (1.23-1.90)	<0.001	1.55 (1.25-1.92)	<0.001	1.52 (1.22-1.89)	<0.001	1.52 (1.22-1.89)	<0.001	1.52 (1.22-1.89)	<0.001	1.53 (1.23-1.90)	<0.001
Indeterminate	0.98 (0.81-1.18)	0.81	0.99 (0.82-1.20)	0.93	0.98 (0.81-1.19)	0.86	0.98 (0.81-1.19)	0.83	0.98 (0.81-1.19)	0.82	0.98 (0.81-1.19)	0.85
Comorbidities												
Cancer	1.31 (1.13-1.52)	<0.001	1.30 (1.12-1.50)	0.001	1.31 (1.13-1.52)	<0.001	1.31 (1.13-1.52)	<0.001	1.32 (1.13-1.53)	<0.001	1.31 (1.13-1.52)	<0.001
Chronic Kidney Disease	1.38 (1.16-1.63)	<0.001	1.37 (1.16-1.63)	<0.001	1.38 (1.16-1.63)	<0.001	1.37 (1.16-1.63)	<0.001	1.38 (1.16-1.64)	<0.001	1.37 (1.16-1.62)	<0.001
Chronic Lung Disease	1.43 (1.25-1.64)	<0.001	1.43 (1.25-1.63)	<0.001	1.44 (1.25-1.64)	<0.001	1.44 (1.26-1.65)	<0.001	1.46 (1.28-1.67)	<0.001	1.42 (1.25-1.63)	<0.001
Dementia	1.49 (1.18-1.88)	0.001	1.51 (1.20-1.90)	0.001	1.48 (1.16-1.87)	0.001	1.54 (1.22-1.95)	<0.001	1.49 (1.17-1.88)	0.001	1.53 (1.22-1.93)	<0.001
Diabetes	1.53 (1.33-1.76)	<0.001	1.54 (1.34-1.77)	<0.001	1.53 (1.33-1.76)	<0.001	1.53 (1.33-1.76)	<0.001	1.52 (1.32-1.76)	<0.001	1.53 (1.33-1.77)	<0.001
Heart Disease	1.40 (1.21-1.63)	<0.001	1.40 (1.21-1.61)	<0.001	1.40 (1.21-1.63)	<0.001	1.40 (1.21-1.62)	<0.001	1.41 (1.22-1.63)	<0.001	1.39 (1.20-1.61)	<0.001
HIV	1.66 (0.94-2.94)	0.08	1.63 (0.93-2.86)	0.09	1.64 (0.93-2.89)	0.08	1.74 (0.98-3.07)	0.06	1.76 (0.99-3.11)	0.05	1.66 (0.94-2.94)	0.08
Liver Disease	1.35 (1.06-1.72)	0.02	1.38 (1.08-1.75)	0.009	1.36 (1.07-1.74)	0.01	1.33 (1.04-1.69)	0.02	1.33 (1.04-1.70)	0.02	1.36 (1.07-1.72)	0.01

© 2023 Wong MS et al. *JAMA Network Open*.

Overweight or Obese[a]	0.66 (0.57-0.77)	<0.001	0.68 (0.59-0.79)	<0.001	0.66 (0.57-0.77)	<0.001	0.67 (0.58-0.79)	<0.001	0.66 (0.57-0.77)	<0.001	0.67 (0.58-0.79)	<0.001
Pregnancy[a]	--		--		--		--		--		--	
Sickle Cell	2.28 (0.41-12.60)	0.35	2.07 (0.39-11.08)	0.34	2.09 (0.39-11.23)	0.39	1.87 (0.34-10.40)	0.48	1.99 (0.35-11.31)	0.44	2.02 (0.37-11.14)	0.42
Ischemic Stroke	1.17 (0.91-1.51)	0.23	1.18 (0.92-1.53)	0.20	1.16 (0.90-1.51)	0.25	1.19 (0.92-1.53)	0.18	1.18 (0.91-1.52)	0.22	1.19 (0.93-1.54)	0.17
Cerebrovascular Disease	1.10 (0.75-1.63)	0.63	1.05 (0.71-1.56)	0.80	1.08 (0.72-1.60)	0.71	1.07 (0.72-1.58)	0.74	1.10 (0.74-1.62)	0.65	1.08 (0.73-1.58)	0.71
Alcohol or Drug Disorder	0.96 (0.82-1.12)	0.58	0.96 (0.82-1.11)	0.56	0.96 (0.82-1.12)	0.60	0.97 (0.83-1.13)	0.72	0.97 (0.83-1.13)	0.69	0.96 (0.82-1.12)	0.58
Vaccine status before COVID-19 Infection[b]												
Not Fully	Ref		Ref		Ref		Ref		Ref		Ref	
Fully vaccinated[c]	0.63 (0.49-0.82)	<0.001	0.63 (0.49-0.80)	<0.001	0.62 (0.48-0.80)	<0.001	0.63 (0.49-0.81)	<0.001	0.61 (0.47-0.79)	<0.001	0.64 (0.50-0.82)	0.001
COVID-19 Time Period												
Early	Ref		Ref		Ref		Ref		Ref		Ref	
Summer Surge	0.63 (0.42-0.95)	0.03	0.61 (0.41-0.92)	0.02	0.65 (0.43-0.98)	0.04	0.58 (0.39-0.87)	0.009	0.64 (0.43-0.97)	0.03	0.59 (0.40-0.88)	0.01
Fall Drop	0.57 (0.38-0.86)	0.007	0.56 (0.37-0.85)	0.006	0.59 (0.39-0.89)	0.01	0.53 (0.36-0.80)	0.003	0.58 (0.38-0.87)	0.009	0.54 (0.36-0.81)	0.003
Winter Surge	0.41 (0.29-0.58)	<0.001	0.40 (0.28-0.57)	<0.001	0.42 (0.29-0.60)	<0.001	0.39 (0.27-0.54)	<0.001	0.42 (0.30-0.59)	<0.001	0.38 (0.27-0.54)	<0.001
Vaccine Drop	0.43 (0.28-0.66)	<0.001	0.45 (0.30-0.68)	<0.001	0.45 (0.30-0.69)	<0.001	0.44 (0.29-0.66)	<0.001	0.47 (0.31-0.71)	<0.001	0.42 (0.28-0.64)	<0.001
Delta Surge	0.47 (0.31-0.70)	<0.001	0.47 (0.32-0.70)	<0.001	0.49 (0.33-0.73)	0.001	0.47 (0.32-0.69)	<0.001	0.51 (0.34-0.76)	0.001	0.45 (0.30-0.66)	<0.001

Notes: a. at time of COVID-19 positive test b. individual who got vaccinated after getting COVID-19 were included as a separate group, but results are not shown; c. fully vaccinated = 2 mRNA vaccines or 1 adenovirus vaccine; -- not calculated due to empty cells ; ORs for HPI percentile and SVI are per 10 percentile change. HPI percentile has been reverse coded (indicated with label lower HPI percentile) such that OR<1 indicates lower risk of hospitalization and OR>1 indicates greater risk of hospitalization; COVID-19 Time periods correspond to the following dates: Early (March - May 2020); Summer surge (June - July 2020); Fall drop (Aug - Oct 2020); winter surge (Nov 2020 - Feb 2021); Vaccine drop (March - June 2021); Delta Surge (July - Oct 2021)

© 2023 Wong MS et al. *JAMA Network Open*.
