# Supplementary material for: Inclusion of Race and Ethnicity With Neighborhood Socioeconomic Deprivation When Assessing COVID-19 Hospitalization Risk Among California Veterans Health Administration Users
Source: JAMA Netw Open. 2023 Mar 3;6(3):e231471. doi: 10.1001/jamanetworkopen.2023.1471 (PMC9984969; doi:10.1001/jamanetworkopen.2023.1471)
Supplement: Supplement 2. — Data Sharing Statement [file jamanetwopen-e231471-s002.pdf]

Data Sharing Statement

Wong. Inclusion of Race and Ethnicity With Neighborhood Socioeconomic Deprivation When Assessing COVID-19 Hospitalization Risk Among California Veterans Health Administration Users. *JAMA Netw Open*. Published March 03, 2023.
doi:10.1001/jamanetworkopen.2023.1471

Data
Data available: No
